# Identifying Predictors of Smoking Switching Behaviours Among Adult Smokers in the United States: A Machine Learning Approach

**DOI:** 10.7759/cureus.69183

**Published:** 2024-09-11

**Authors:** Yue Cao, Xuxi Zhang, Ian M Fearon, Jiaxuan Li, Xi Chen, Fangzhen Zheng, Jianqiang Zhang, Xinying Sun, Xiaona Liu

**Affiliations:** 1 Department of Health Sciences, Smoore Research Institute, Shenzen, CHN; 2 School of Public Health, Peking University, Beijing, CHN; 3 Department of Scientific Research, whatIF? Consulting Ltd, Harwell, GBR

**Keywords:** cigarette smoking, e-cigarettes, machine learning, population assessment of tobacco and health study, switching behaviour

## Abstract

Completely abstaining from cigarette smoking or fully switching to e-cigarette (EC) use may be beneficial for reducing the global burden of smoking-related diseases. This study aimed to identify and compare the top 10 prospective predictors of smokers switching away from smoking in the United States. Data from adult exclusive cigarette smokers at Wave 4 of the Population Assessment of Tobacco and Health (PATH) study, who were followed up at Wave 6, were analysed. An Xgboost-based machine learning (ML) approach with a nested cross-validation scheme was utilised to develop a multiclass predictive model to classify smokers' behavioural changes from W4 to W6, including smoking cessation, full and partial switching to EC, and cigarette non-switching. The SHapley Additive exPlanations (SHAP) algorithm was deployed to interpret the top 10 predictors of each switching behaviour. A total of 396 variables were selected to generate the four-class prediction model, which demonstrated a micro- and macro-average area under the receiver operating characteristics curve (ROC-AUC) of 0.91 and 0.81, respectively. The top three predictors of smoking cessation were prior regular EC use, age, and household rules about non-combusted tobacco. For full switching to EC use, the leading predictors were age, type of living space, and frequency of social media visits. For partial switching to EC use, the key predictors were daily cigarette consumption, the time from waking up to smoking the first cigarette, and living with tobacco users. ML is a promising technique for providing comprehensive insights into predicting smokers' behavioural changes. Public health interventions aimed at helping adults switch away from smoking should consider the predictors identified in this study.

## Introduction

Cigarette smoking has remained the world’s leading cause of preventable morbidity and mortality for decades [1]. The World Health Organization has recognised smoking as a major contributor to the global prevalence of non-communicable diseases, such as cardiovascular disease, stroke, cancer, and emphysema. It is estimated that by 2030, tobacco-related illnesses will claim the lives of over 8 million smokers annually [2].

The harmful effects of smoking result from long-term exposure to thousands of hazardous chemicals and carcinogens released through the combustion of tobacco [3]. There is a consensus that smoking cessation is the most effective approach to preventing smoking-related diseases [4]. However, for individuals who are unable or unwilling to quit smoking, alternative tobacco harm reduction (THR) options, such as switching to non-combustible products like electronic cigarettes (ECs), may help reduce both individual and population-level disease risk [5]. ECs entered the global market in the mid-2000s [6], and since that time, the world’s tobacco and nicotine product landscape has changed dramatically. ECs deliver nicotine to users but with substantially lower levels of exposure to toxicants compared to combustible cigarette smoking [7,8], leading public health bodies, such as Public Health England, to suggest that EC use may be at least 95% less harmful than smoking [9]. Smoking reduction, whether through completely switching to EC use or partially switching to EC use (i.e., dual use of cigarettes and ECs), may decrease the risk of developing smoking-related diseases among adult smokers [10,11]. However, other evidence has shown that the extent of change in toxicant exposure among dual users was impacted by their change in the number of cigarettes smoked per day (CPD), with a reduction of over 50% in CPD yielding positive short-term health outcomes, and an increase of over 50% in CPD leading to negative outcomes [12].

Research aiming to detect factors associated with switching behaviours has shown a high degree of variability in study designs and reported results. Demographic characteristics, including age, sex, race/ethnicity, sexual orientation, educational attainment, and poverty level, as well as tobacco use characteristics, including total number of products used, nicotine dependence, and type and frequency of product used, have been identified as significant predictors of transitions in tobacco product use over a one-year period [13]. Past evidence suggests that individual relative risk perception of ECs compared to cigarettes has a strong and consistent association with aspects or stages of switching from smoking to vaping among smokers regardless of their history of EC use [14]. Additionally, willingness to vape for harm reduction and smoking cessation purposes played a crucial role in achieving subsequent smoking abstinence [15]. Environmental factors, especially peer and social influences, can also lead to EC initiation. A systematic review has determined that exposure to advertising increased the likelihood of EC uptake among adult smokers [16]. Another systematic review has identified 14 indicators of quitting success, which could be grouped into four categories: individual health status, healthcare setting, tobacco smoking variables, and intervention characteristics [17].

Health-promoting behaviours such as switching to harm-reduced tobacco products are complex and multifaceted, and different theories and models have been developed to understand why people undergo these behaviour changes. Theories such as Health Belief Models (HBM) [18], Theory of Planned Behaviour (TPB) [19], and Social Cognitive Learning Theory (SCLT) [20], have attempted to explain people’s engagement in health-related behaviours through the interplay between individual, social, environmental, economic, and institutional factors [21]. However, previous studies on smoking switching behaviours have primarily focused on a restricted set of pre-specified determinants, without fully considering factors across a broad spectrum of dimensions. Furthermore, these studies have neglected the comparison of diverse predictors for various behavioural changes, thus hindering a comprehensive understanding of the facilitators and barriers associated with smoking transition patterns.

Machine learning (ML) models utilising tree-based algorithms have recently emerged in the field of THR research and started gaining popularity [22]. ML techniques are well known for their ability to accommodate multiple predictors and unravel complicated non-linear associations between predictors and outcomes. Risk or protective factors for tobacco use-related behaviours have been widely studied by ML models in recent years [22-26]. However, little attention has been given to investigating predictors associated with tobacco use behaviours other than the initiation of non-combustible tobacco product use, such as ECs or smokeless tobacco products, among adolescents or young adults, or complete smoking cessation among adult smokers. To the best of our knowledge, no study has been conducted to explore long-term predictors of different switching behaviours among adult smokers using ML techniques. Therefore, this study aimed to use ML techniques not only to develop a predictive model but also to identify and compare the top 10 predictors of different switching behaviours among adult smokers. These included complete smoking cessation, full or partial switching to EC use, and non-switching, i.e., remaining exclusively smoking cigarettes. Identifying predictors of such switching behaviours can provide a deeper understanding of smokers’ future likelihood of different behavioural changes and facilitate a greater understanding of how to support THR.

## Materials and methods

Data and study sample

The Population Assessment of Tobacco and Health (PATH) study is an ongoing, nationally representative, longitudinal cohort study that examines tobacco use behaviours and their effects on population health in the United States (US). The study currently consists of six publicly available waves: Wave 1 (data collected from September 2013 to December 2014), Wave 2 (October 2014 to October 2015), Wave 3 (October 2015 to October 2016), Wave 4 (December 2016 to January 2018), Wave 5 (December 2018 to November 2019), and Wave 6 (March 2021 to November 2021). Detailed information regarding the survey design and recruitment process for the PATH study has been previously reported [27]. To identify prospective predictors of smokers’ switching behaviours, we included a total of 5,039 respondents aged over 18 years, who were established exclusive smokers at Wave 4 and followed up at Wave 6 of the PATH Study.

Study variables

Participants were considered established exclusive smokers for a given wave if they had smoked at least 100 cigarettes in their lifetime without concurrently using any EC products. Similarly, they were characterised as established exclusive EC users if they had used ECs fairly regularly in their lifetime without concurrently smoking cigarettes. The primary outcome of the study was the uptake of different switching behaviours at Wave 6. Switching behaviour was defined as a four-class variable as follows: (1) smoking cessation: completely abstained from smoking; (2) full switching to EC use: switched to established exclusive EC use; (3) partial switching to EC use: switched to dual use of cigarettes and ECs (both established); and (4) cigarette non-switching: continued exclusively smoking cigarettes.

As described elsewhere [24], susceptibility to EC use was determined by reporting “Definitely yes,” “Probably yes,” or “Probably not” to the following questions: “Think you will use an electronic nicotine product in the next year,” “Think you will try an electronic nicotine product soon,” “Would try an electronic nicotine product if one of your best friends offered it to you,” and “A little curious,” “Somewhat curious,” or “Very curious” to the question “Ever been curious about using an electronic nicotine product.” Non-susceptible adult smokers answered “Definitely not” to the first three questions and “Not at all curious” to the last question. Susceptibility to hookah, snus, and smokeless tobacco were determined with similar questions.

Variables indicating computerised randomisation of the question order (n = 108), variables missing for at least 30% of the study sample (n = 1,489), variables with extremely low variances (i.e., responses that were the same in 99% of respondents, n = 182), and variables used to derive or highly correlated with other variables were removed as they could not provide sufficient or independent information (n = 54) for the final predictive model. After pre-processing the data, 396 variables out of 2,229 from the original dataset were available for the subsequent model development.

Model development and statistical analysis

Nested cross-validation (CV) [28] was performed, with an outer repeated 10-fold CV for model evaluation and an inner 10-fold CV for hyper-parameter optimisation. Xgboost [29] classifiers were established at each step of the inner CV. Xgboost can capture non-linear associations and interaction effects between variables and the outcome, as well as flexibly handling missing values and multilevel categorical variables. Hyper-parameters were chosen to maximise balanced accuracy (BACC) across all validation sets using the Bayesian Optimisation Algorithm. An Xgboost classifier was trained on the whole outer training set with the hyper-parameters found during the inner CV. To produce an unbiased estimate of model performance, area under the receiver operating characteristics curves (ROC-AUC) and area under the precision-recall curve (PR-AUC) were calculated on each outer fold test data, pooled together to determine prediction accuracy.

We then created a final multiclass predictive model with Xgboost on the full dataset with the optimal hyperparameters tuned using 10-fold CV. The SHapley Additive exPlanations (SHAP) [30] algorithm was deployed to extract the 10 most influential variables associated with the switching behaviours of interest. Positive SHAP values indicated an increase in the probability of undergoing each switching behaviour, while negative SHAP values represented a declining probability. The SHAP dependence plots, stratified by smoking or EC use status, were plotted to thoroughly explain and compare the relationships between significant variables and different switching behaviours. All statistical analyses were conducted using R version 4.3.0 (R Foundation, Vienna, Austria).

## Results

Sociodemographic characteristics

Sociodemographic characteristics of PATH survey respondents included in our analyses are presented in Table 1. Among the respondents, 18.5% (n = 938) completely abstained from smoking, 4.4% (n = 232) switched to exclusively using ECs, 7.2% (n = 394) became dual users of cigarettes and ECs, and the majority (69.9%, n = 3,475) remained exclusively smoking. The population-weighted proportions of males, individuals aged 25 years or older, White individuals, non-Hispanic individuals, those with less than a high school education, married individuals, and those in low-income groups were 53.4%, 92%, 77.1%, 87%, 57.6%, 36.2%, and 72.4%, respectively. Statistically significant differences were observed among switching behaviours when examining gender, age, race, ethnicity, marital status, education level, and annual household income (all p-values < 0.05).

**Table 1:** Sociodemographic characteristics of respondents stratified by switching behaviours in the US PATH study. ^a^Statistics were generated using Pearson’s chi-squared test with Rao-Scott second-order correction for a difference between switching behaviours. * p<0.05, ** p<0.01, *** p<.001. ^b^Other races include respondents who are American Indian or Alaska Native, Asian Indian, Chinese, Filipino, Japanese, Korean, Vietnamese, Other Asian, Native Hawaiian, Guamanian or Chamorro, Samoan, Other Pacific Islander and multiracial. EC, electronic cigarette; US, United States; PATH, Population Assessment of Tobacco and Health.

Baseline characteristics	Switching behaviour				Total (N = 5,039) n (weighted %)	Statistics^a^
	Smoking cessation (N = 938) n (weighted %)	Full switching to EC (N = 232) n (weighted %)	Partial switching to EC (N = 394) n (weighted %)	Non-switching (N = 3,475) n (weighted %)		
Gender						15.245^*^
Male	464 (54.4)	103 (45.9)	155 (45.8)	1,627 (54.4)	2,349 (53.4)	
Female	474 (45.6)	129 (54.1)	239 (54.2)	1,848 (45.6)	2,690 (46.6)	
Age						337.800^***^
18 to 24 years old	137 (9.5)	89 (24.6)	114 (20.5)	299 (5.2)	639 (8.0)	
25 to 44 years old	433 (47.6)	112 (59.3)	197 (57.1)	1,393 (41.7)	2,135 (44.7)	
45 or more years old	368 (42.9)	31 (16.1)	83 (22.4)	1,783 (53.1)	2,265 (47.3)	
Race						24.234^**^
White	684 (77.8)	184 (84.8)	307 (81.2)	2,473 (75.9)	3,648 (77.1)	
Black	152 (14.5)	22 (8.3)	46 (9.6)	712 (16.7)	932 (15.4)	
Other^b^	102 (7.7)	26 (6.9)	41 (9.2)	290 (7.4)	459 (7.5)	
Hispanic						28.157^***^
Yes	179 (17.8)	35 (9.1)	39 (9.2)	443 (12.4)	696 (13.0)	
No	759 (82.2)	197 (90.9)	355 (90.8)	3,032 (87.6)	4,343 (87.0)	
Marital status						56.209^***^
Married	322 (36.6)	67 (32.4)	96 (27.5)	1,213 (37.2)	1,698 (36.2)	
Widowed, divorced, or separated	237 (25.2)	36 (22.0)	101 (28.3)	1,079 (31.5)	1,453 (29.7)	
Never married	374 (38.2)	128 (45.6)	197 (44.2)	1,170 (31.3)	1,869 (34.1)	
Education level						96.352^***^
High school graduate or less	415 (46.2)	117 (52.2)	212 (55.3)	1,986 (61.2)	2,730 (57.6)	
Some college (no degree) or associate degree	361 (36.7)	87 (38.7)	148 (37.6)	1,126 (29.3)	1,722 (31.7)	
Bachelor's degree or advanced degree	156 (17.1)	28 (9.1)	32 (7.1)	347 (9.5)	563 (10.7)	
Household income						21.968^***^
Less than $50,000	632 (66.9)	161 (67.9)	288 (75.4)	2,563 (73.9)	3,644 (72.4)	
$50,000 or more	274 (33.1)	65 (32.1)	86 (24.6)	751 (26.1)	1,176 (27.6)	

Model performance

**Figure 1:**
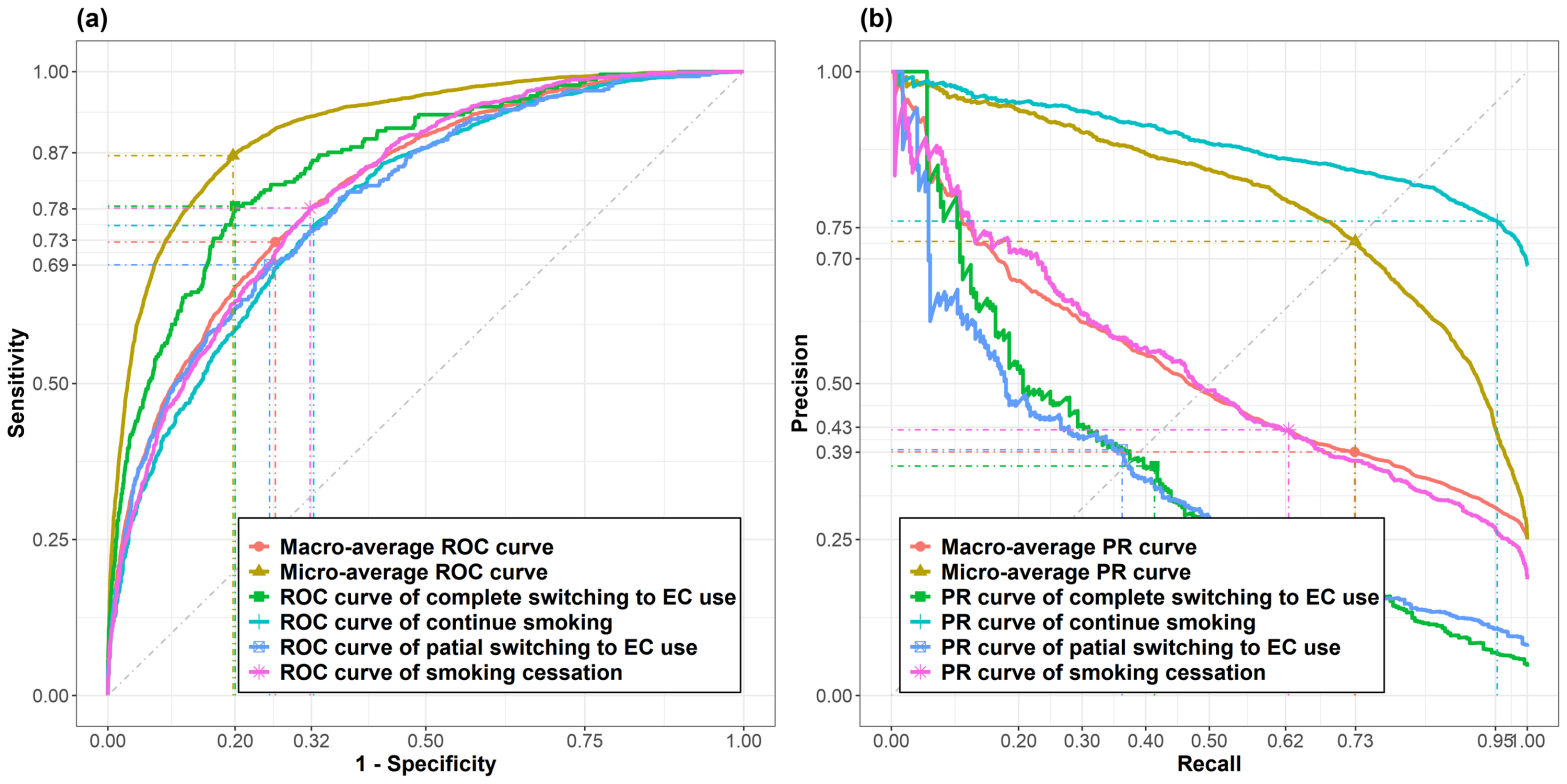
Receiver operating characteristics (ROC) (a) and precision-recall (PR) curves (b) of Xgboost-based machine learning model evaluated by nested cross-validation scheme using one-vs-the-rest (OvR) multiclass strategy. Model classes included smoking cessation, full switching to EC use, partial switching to EC use, and non-switching. EC, e-cigarette.

To assess model performance and ensure unbiased generalisability, ROC-AUC and PR-AUC were calculated for each class (one-vs-rest) by pooling results from the nested CV scheme (Figure 1). The four-class prediction model had a micro- and macro-average ROC-AUC of 0.91 and 0.81, and a micro- and macro-average PR-AUC of 0.79 and 0.52, respectively. The ROC-AUC values for the categories of identification were 0.81, 0.86, 0.80, and 0.79 for smoking cessation, full switching to ECs, partial switching to ECs, and continuation of smoking, respectively. The PR-AUC values were 0.52, 0.35, 0.33, and 0.88 for smoking cessation, full switching to ECs, partial switching to ECs, and cigarette non-switching, respectively. The overall and class-specific classification performances demonstrated good discriminative power of the model in predicting switching behaviours among the adult population.

The most influential predictors of switching behaviours

**Figure 2:**
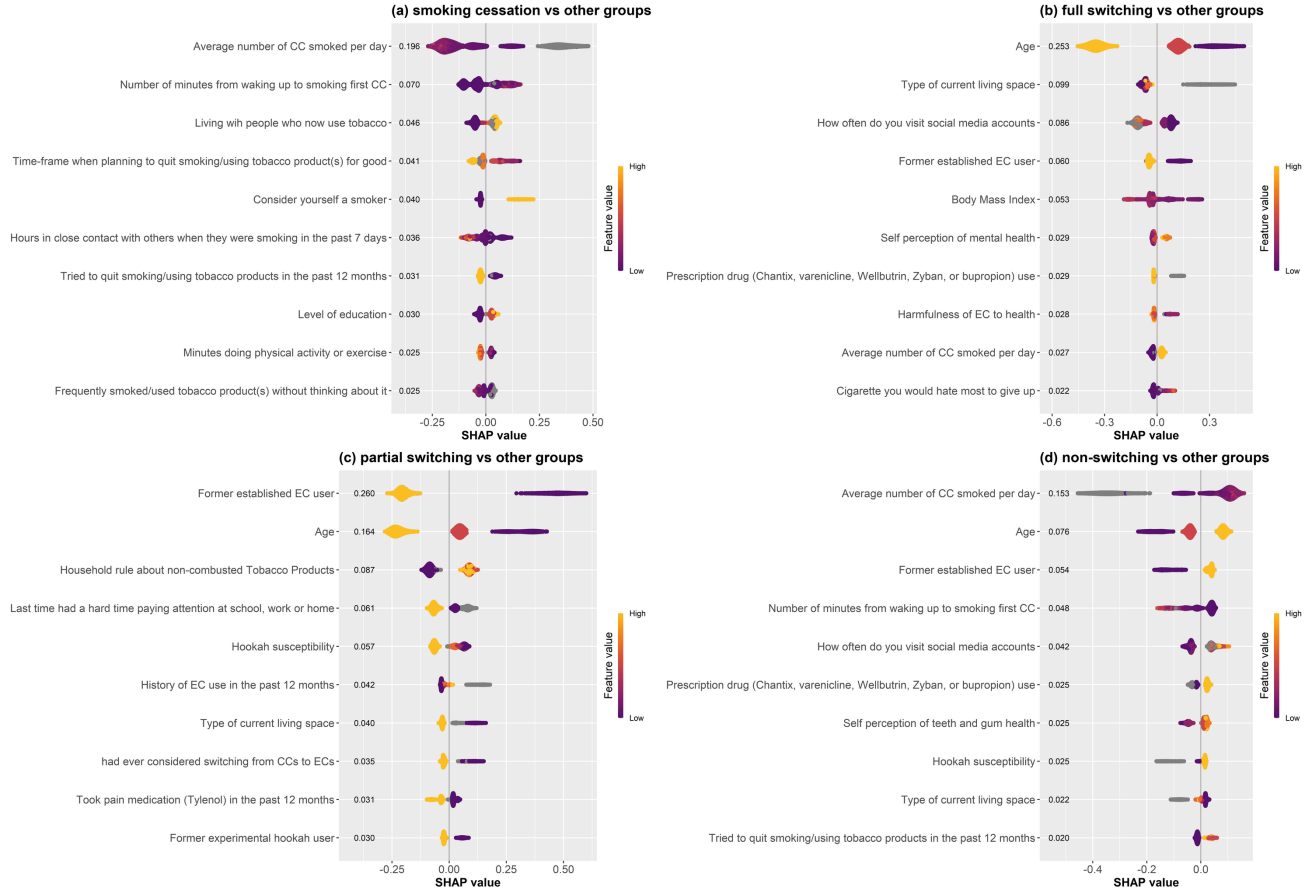
The most influential variables predicting smokers' switching behaviours. Switching behaviours assessed were (a) smoking cessation, (b) full switching to EC use, (c) partial switching to EC use, and (d) non-switching. The importance of predictors was ranked by the mean of the absolute of their SHAP values and listed in descending order. Positive SHAP values indicate an increased probability of that specific switching behaviour while negative SHAP values indicate a decreased probability. CC, combustible cigarette; EC, e-cigarette; SHAP, SHapley Addictive exPlanations.

The 10 most influential predictors of smoking cessation, with the highest mean absolute SHAP values, are shown in Figure 2a. These predictors included the average CPD, the number of minutes from waking up to smoking the first cigarette, living with tobacco users, the time frame to quit smoking/using tobacco products, self-identity as a smoker, the number of hours spent closely with others when they were smoking, an attempt to quit smoking/using tobacco in the past 12 months, education level, length of exercise time, and the frequency of smoking/using tobacco products without thinking about it.

The 10 most influential predictors of full switching to EC use, with the highest mean absolute SHAP values, are shown in Figure 2b. These predictors included age, type of current living space, frequency of social media visits, history of regular EC use, body mass index, self-perception of mental health, use of quit-smoking medications (e.g., Chantix, varenicline, Wellbutrin, Zyban, or bupropion), perception of the harmfulness of ECs to health, average CPD, and the cigarette that would be most difficult to give up.

The 10 most influential predictors of partial switching to EC use, with the highest mean absolute SHAP values, are shown in Figure 2c. These predictors included history of regular EC use, age, household rules about non-combusted tobacco product use, difficulty paying attention at school, work, or home in the past 12 months, hookah susceptibility, history of EC use in the past 12 months, type of current living space, past consideration of switching from smoking to EC use, use of anti-inflammatory or pain medication (e.g., Tylenol) in the past 12 months, and experimental (non-regular) history of hookah use.

The 10 most influential predictors of cigarette non-switching, with the highest mean absolute SHAP values, are shown in Figure 2d. These predictors included the average CPD, age, history of regular EC use, number of minutes from waking up to smoking the first cigarette, frequency of social media use, use of quit-smoking medications (e.g., Chantix, varenicline, Wellbutrin, Zyban, or bupropion), self-perception of dental health (teeth and gums), hookah susceptibility, type of current living space, and any attempts to quit smoking/using tobacco in the past 12 months.

**Figure 3:**
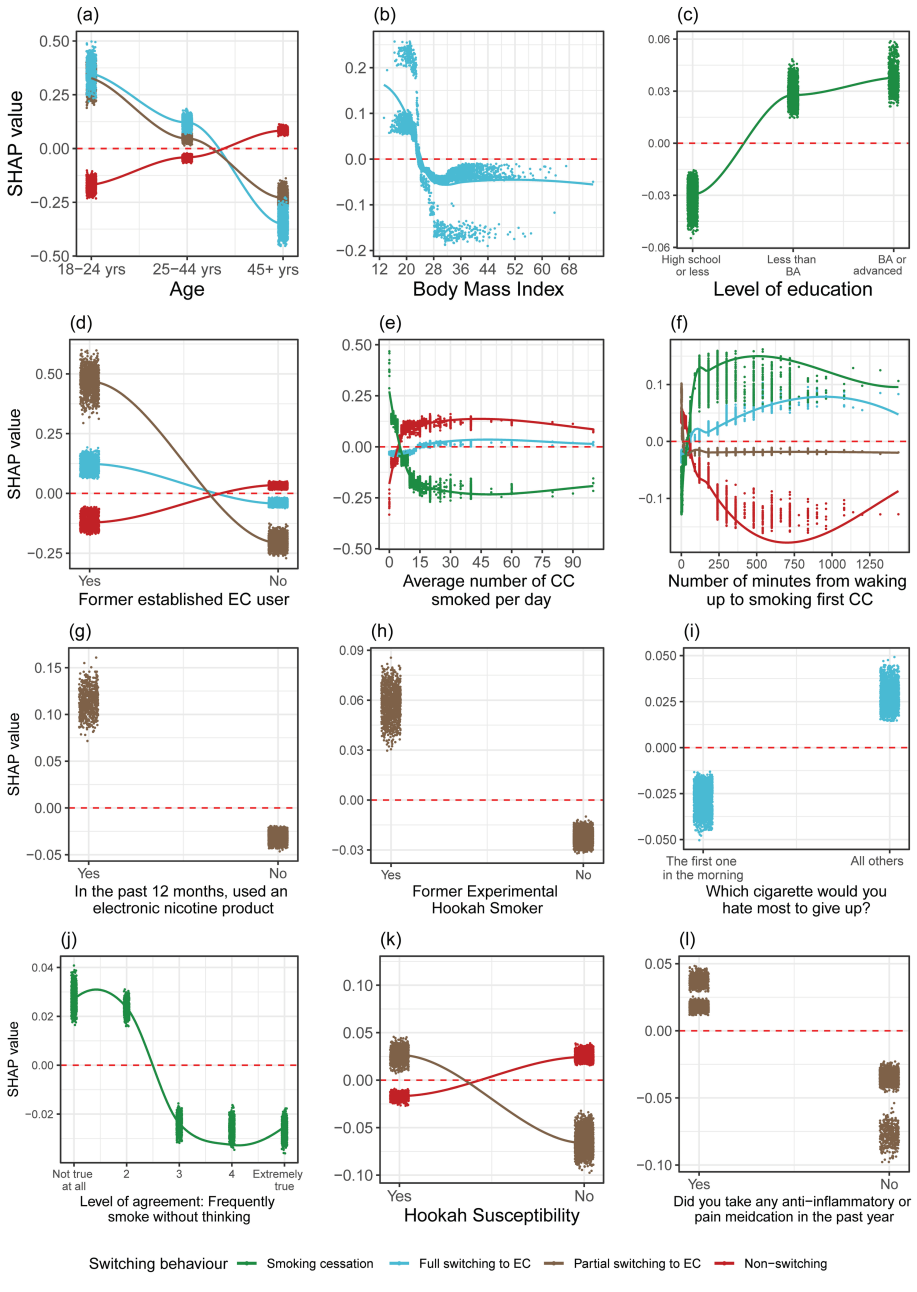
SHP feature dependence plots of the identified variables on sociodemographic characteristics, tobacco dependence, tobacco/nicotine use, and susceptibility for predicting switching behaviours. SHAP values are presented on the y-axis, and variables are presented on the x-axis. Positive SHAP values indicate increased probability of that specific switching behaviour while negative SHAP values indicate decreased probability. Variables presented in the panels are (a) age, (b) body mass index, (c) level of education, (d) former established EC user, (e) average number of CCs smoked per day, (f) number of minutes from waking to smoking first CC, (g) used an electronic nicotine product in the past 12 months, (h) former experimental hookah user, (i) which cigarette they would most hate to give up, (j) level of agreement with frequently smoking without thinking about it, (k) susceptibility to hookah use, and (l) used any anti-inflammatory or pain medication in the past year. EC, e-cigarette; CC, combustible cigarette; SHAP, SHapley Addictive exPlanations.

**Figure 4:**
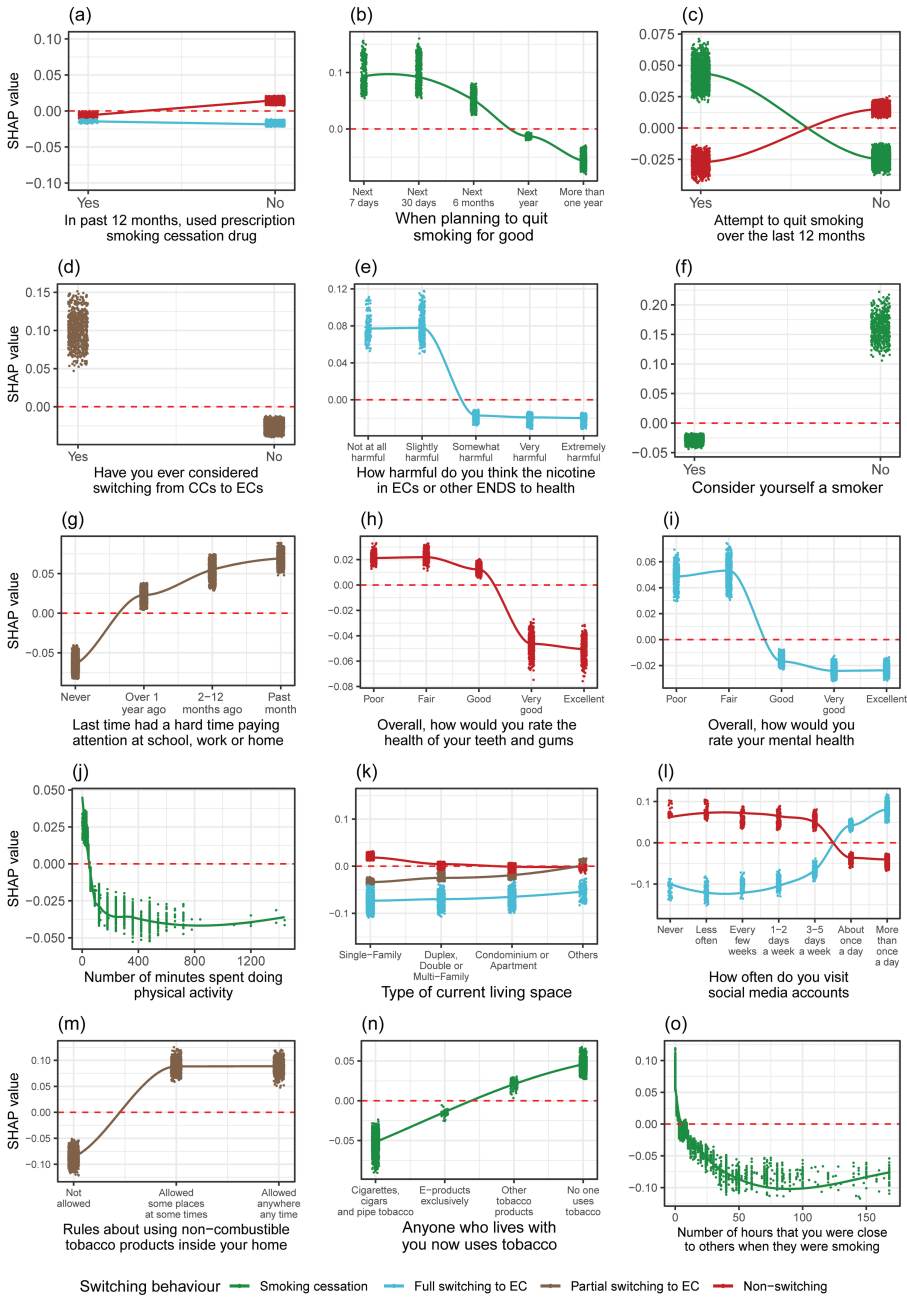
SHAP feature dependence plots of the identified variables on environmental factors, health status and lifestyle, self-efficacy factors, and risk perception for predicting smoking switching behaviours. SHAP values are presented on the y-axis, and variables are presented on the x-axis. Positive SHAP values indicate increased probability of that specific switching behaviour while negative SHAP values indicate decreased probability. Variables presented in the panels are (a) used a prescription smoking cessation drug in the past 12 months, (b) when they plan to quit smoking for good, (c) whether they have tried to quit smoking in the past 12 months, (d) if they have considered switching from CCs to ECs, (e) perceptions of the harmfulness to health of the nicotine in ECs or ENDS, (f) whether they consider themselves a smoker, (g) the last time they had a hard time paying attention at school, work or home, (h) self-rated overall health of teeth and gums, (i) self-rated overall mental health including stress, depression and problems with emotions, (j) number of minutes spent doing physical activity or exercise of at least moderate intensity, (k) type of current living space, (l) how often they visit social media accounts, (m) rules concerning using non-combustible tobacco products inside their home, (n) anyone who lives with them now who uses tobacco, and (o) the number of hours that they were in close contact with others when they were smoking. EC, e-cigarette; CC, combustible cigarette; ENDS, electronic nicotine delivery system(s); SHAP, SHapley Addictive exPlanations.

In total, 27 prospective predictors most influentially associated with smokers' switching behaviours were identified (Figures 3, 4). These predictors could be grouped into seven categories, which are described as follows.

Sociodemographic characteristics

Younger adult smokers (18-44 years old) exhibited a higher likelihood of partially or fully switching to using ECs, while older individuals (45 or more years old) were more inclined to continue smoking exclusively. Compared to those with a body mass index (BMI) of 25 kg/m² or higher, adult smokers with a BMI lower than 25 kg/m² were positively associated with completely switching to using ECs. Education levels beyond high school graduation were associated with positive SHAP values, indicating an increased likelihood of smoking cessation.

Dependence

Former established EC users (i.e., those who had used ECs fairly regularly 12 months ago) were associated with positive SHAP values for undergoing either a partial or full switch to EC use, but negative SHAP values for continuing smoking cigarettes exclusively. Past 12-month use of ECs and hookah was positively associated with a tendency to become dual users of cigarettes and ECs. The greater the average CPD, the longer the time interval from waking up to smoking the first cigarette, the more likely smokers were to continue smoking exclusively and the less likely they were to quit smoking in the future. Smokers who did not have a strong aversion to giving up their first cigarette after waking up were more likely to completely switch to EC use. Conversely, individuals who frequently smoked without even thinking about it were less likely to achieve smoking cessation.

Substance use and susceptibility to tobacco

Susceptibility to hookah was associated with positive SHAP values for partial switching to ECs, and negative SHAP values for continuation of smoking cigarettes. Also, reporting taking pain medication (particularly Tylenol) in the past 12 months was associated with a higher probability of transitioning to dual use of cigarettes and ECs among adult smokers.

Self-efficacy

Individuals who had ever considered switching from smoking to using ECs were more likely to switch to dual use during the follow-up period. Exclusive cigarette smokers who did not attempt to quit smoking or other tobacco products in the past 12 months were more likely to continue smoking, while individuals who tried to quit smoking over the past year were more likely to undergo complete smoking cessation. Moreover, having a quitting plan in the near future was associated with positive SHAP values for subsequent smoking cessation behaviour among adult smokers.

Health status and lifestyle

Self-perception of good teeth and gum health was found to deter smokers from continuing exclusive cigarette smoking. For smokers who fully switched to exclusive EC use, self-perception of worse mental health was another driving factor. Individuals who reported having difficulty paying attention at school, work, or home recently were more likely to become dual users at a later stage. Furthermore, respondents who spent less than approximately 60 minutes doing physical activity or exercise of at least moderate intensity were more likely to engage in smoking cessation compared to those who exercised more than 60 minutes.

Risk perception and self-perception

Compared to those who perceived EC as somewhat to extremely harmful to their health, respondents who perceived EC as not at all harmful or slightly harmful were more likely to switch completely to EC use. Additionally, smokers who did not identify themselves as smokers were more likely to abstain from smoking over time.

Social and environmental influences

Living in a single-family home increased the likelihood of continuing to smoke cigarettes exclusively while decreasing the probability of partially or fully switching to EC use. The frequency of social media use also had an impact on smokers' behaviour. Higher exposure to social media was associated with a greater likelihood of switching to exclusive EC use, while less frequent exposure was linked to a higher probability of continuing to smoke exclusively. Among dual users, one possible motivator for concurrent smoking and vaping could be living in a place where non-combustible tobacco products are allowed. Not living with tobacco users and spending fewer hours in close contact with others while they were smoking were positively associated with smoking cessation.

## Discussion

This study identified prospective predictors of different switching behaviours among a nationally representative sample of adult smokers in the US. Our analysis encompassed a wide range of potential variables and successfully detected key factors across multiple dimensions, including sociodemographic characteristics, dependence, substance use and susceptibility, social and environmental influences, self-efficacy, health status and lifestyle, as well as risk and self-perceptions. These findings provide valuable insights into the dynamics of smoking behaviours and switching patterns among US adults.

Several predictors we identified for smoking cessation aligned with previous literature [17]. Specifically, our findings indicated that sociodemographic characteristics (such as educational attainment), nicotine dependence (including current daily cigarette consumption), cohabitation and social network factors (such as living with tobacco users), and self-efficacy factors (e.g., motivation to quit or quitting history) were associated with smoking cessation. Additionally, our observations suggested that phantom smokers, defined as those who are currently smoking but give a negative response to “Do you consider yourself a smoker” [31], were more likely to abstain from smoking compared to self-identified smokers. Other evidence also showed that phantom smokers expressed more confidence in their ability to quit and experienced less pressure to change their smoking behaviours [31,32]. More studies are needed to understand the underlying mechanism between this ‘phantom’ smoking status and subsequent quitting success. Moreover, we found that shorter durations of moderate to intense exercise (less than 60 minutes per day) could facilitate smoking cessation, although the impact of exercise intensity and duration on the success rate of smoking abstinence requires further investigation.

Age emerged as the most influential predictor of fully switching to EC use, which aligns with concerns that ECs are more appealing to younger individuals. We also found that adult smokers with lower BMI were more likely to make a complete switch to EC use. Weight-motivated tobacco use has long been recognised, as nicotine-containing tobacco products can suppress appetite, reduce food cravings, and increase metabolic rate [33]. Similar to smoking abstainers, we found that smokers who were less nicotine dependent had a higher probability of fully switching from smoking to using ECs. Frequent engagement with social media played a crucial role in driving complete switching, potentially due to increased exposure to commercial promotion of ECs, leading to more positive attitudes towards EC use [34]. Furthermore, Yang et al. pointed out that smokers who experienced serious psychological distress had a greater intention to switch to ECs completely [35]. In our study, smokers reporting poor or fair mental health were more inclined to fully switch to EC use as well.

Regarding the transition from exclusive smoking to dual use, nicotine dependence was a significant influencing factor. Our results suggested that being a former established or experimental EC/hookah user enhanced the likelihood of exclusive smokers switching to partial EC use. Susceptibility to hookah use was also found to be influential. Interestingly, we observed a positive effect of past-year pain medication use on partial switching from cigarette smoking to EC use, possibly indicating that these smokers sometimes sought pain relief through nicotine-containing products [24]. A social environment favourable to non-combustible tobacco products could also motivate exclusive cigarette smokers to use cigarettes and ECs concurrently, as previously described [36]. Consistent with evidence that nicotine increased sustained attention [37] and the association between attention deficit hyperactivity disorder (ADHD) diagnosis and higher prevalence of dual or poly use of tobacco products among youth [38], our model reflected that smokers experiencing recent difficulty paying attention at school, work, or home were more likely to partially switch to EC use.

The determinants identified for the continuation of exclusive cigarette smoking were more intuitive. Continued exclusive smokers were older, exhibited higher levels of nicotine dependence, had little experience with EC use, showed no susceptibility to hookah, visited social media less frequently, and had not attempted to quit smoking in the past year. Additionally, we found that self-perception of good or very good teeth and gum health discouraged sustained use of cigarettes, probably out of consideration to maintain their current oral health status. Evidence suggested that young adults who believed that smoking causes oral or periodontal problems were at significantly lower risk of becoming current smokers [39]. Further studies are needed to examine whether self-perception of poor teeth and oral health can influence smokers’ decisions to continue smoking cigarettes or not.

Methodologically, our study provides further evidence of the utility of ML techniques in devising predictive modelling in behavioural changes of adult smokers. One strength of applying ML is that we did not need to predetermine any associations between factors and the primary outcome. Instead, ML allowed us to simultaneously take a large number of variables into account, avoiding overlooking relevant predictors as well as any non-linear relationships with the uptake of different switching behaviours. Another strength of this study was the use of respondents who were nationally representative of adult exclusive smokers in the US. Since ML techniques are highly data-driven, the generalisation of the predictive model is largely dependent on the quality and size of the training sample. Furthermore, the identification of predictors for switching behaviours holds significant implications for public health interventions and policies, promoting the implementation of tailored strategies to support and encourage individuals in reducing harms associated with smoking, and therefore contributing to mitigating tobacco-related morbidity and mortality as well as enhancing public health.

The SCLT [20], an advancement of the Social Learning Theory (SLT), emphasises the interaction between cognitive or personal factors (such as knowledge, expectations, and attitudes), behavioural factors (including individual skills, practice, and self-efficacy), and environmental factors (such as social norms, access in the community, and influence on others) in shaping human behaviour. The theory recognises that human behaviour can be changed or modified by addressing these interconnected aspects. The determinants for each switching behaviour identified in our study strongly match the principles of SCLT. For instance, we found that exclusive smokers who completely switched to EC use were less nicotine dependent, had experience using ECs or quit-smoking medicines, visited social media accounts more frequently, were more aware of poor mental health, and perceived ECs in a more positive way. On the one hand, the heightened self-awareness of mental health and positive attitudes towards ECs are indicative of cognitive/personal factors in the SCLT. Also, active engagement on social media may make knowledge about EC use more accessible to individuals. These characteristics correspond to part of the theory, suggesting that individuals’ knowledge, expectations for health outcomes, and attitudes could collectively influence their behaviour change decisions. On the other hand, prior experience with ECs or quit-smoking medicines may help individuals develop the skills as well as the self-efficacy required to successfully quit smoking by switching to reduced-risk alternatives such as ECs. In addition, the influence of living with family members who also use tobacco/nicotine products reflects environmental factors, suggesting that participants may be more susceptible to adopting new behaviours when taking social norms and the influences of people around them into account. Moving forward, our next step is to investigate the effectiveness of the top identified determinants in predicting the uptake of varied switching behaviours using the SCLT framework.

Limitations

This study has several limitations. First, we extracted data from a publicly available database. The data were based on self-report measures, which may be subject to recall bias or social desirability bias. Second, our analyses were limited to the information collected in the PATH study, and thus we were unable to include other influential predictors related to switching behaviours. Third, we established the ML models based on unweighted PATH data, because ML algorithms such as Xgboost cannot account for the PATH study’s complex design and weights. However, our results still provided valuable insights into predictors of various switching behaviours due to the nationally representative nature of the PATH study, the large sample size, and the longitudinal approach to survey data collection. Lastly, despite the model’s ability to include missing data without special processing, it is difficult to interpret some top variables when the missing values and non-missing values were separated completely by the positive and negative SHAP values.

## Conclusions

In summary, our study extends existing research on applying ML techniques to better predict and understand the multi-dimensional nature of four specific switching behaviours among adult smokers. This implies that ML may be a promising method to predict tobacco-related behaviours with high predictive power and the capacity to discover emerging predictors. Furthermore, with the assistance of SCLT, we can gain an in-depth understanding of the factors that motivate smokers to switch. Identifying the most influential predictors can potentially aid in developing effective interventions and strategies to promote potentially less harmful switching behaviours among target populations, such as smokers, ultimately leading to improved public health outcomes.

## Appendices

Guidance for figure interpretation

Figure 2 presents the 10 most influential predictors for each switching behaviour based on the derived SHAP values. In each plot, the x-axis represents the SHAP value, while the y-axis lists the 10 predictors. Predictors are ranked in order of their importance to the prediction of each behaviour, reflecting how much they influenced the model’s prediction. The importance is determined by the mean of the absolute SHAP values across all individuals, displayed near the y-axis. The higher the mean of the absolute SHAP values, the more significant the predictor is to the predicted outcome. For each primary outcome category, each individual had a SHAP value for a specific predictor. In Figures 2 (a), (b), (c), and (d), each dot symbolizes an individual. The colour of the dot represents the value of the predictor, with relatively high variable values appearing as yellow dots and relatively low variable values appearing as purple dots. The horizontal location of the dot shows the SHAP value for a specific predictor. SHAP values are associated with one’s probability of undergoing a specific behaviour. A positive SHAP value for a predictor suggests that the predictor increased the individual’s likelihood of the predicted behaviour, while a negative SHAP value indicates that the predictor contributed to the individual not performing the behaviour.

SHAP dependence plots (Figures 3, 4) illustrate the relationships between each of the 27 variables and smoking switching behaviours. Different colours represent distinct behaviours: green for smoking cessation, blue for full switching to EC use, dark orange for partial switching to EC use, and red for non-switching. Each plot features varying colour sets, as each predictor ranks among the top 10 most influential predictors for different types of switching behaviours. The x-axes display the value of the predictors, while the y-axes show the corresponding SHAP values. In each plot, each dot represents an individual, and the vertical location of the dot reflects the magnitude of the SHAP value. Individuals (or dots) clustered within the same coloured regions share the same predictor values. The width of the regions was created using geom_jitter (width=0.05) in R to address overplotting due to variable discreteness. Positive SHAP values indicate an increased possibility of executing a certain behaviour, while negative SHAP values indicate a decreased likelihood. We applied the geom_smooth function in R to generate smooth regression lines between variable values and their SHAP values, stratified by switching behaviour. Some plots lack a smooth regression line since the predictor values were too discrete to provide enough information to define a clear regression pattern.
